# Epidemiological and advanced therapeutic approaches to treatment of uveitis in pediatric rheumatic diseases: a systematic review and meta-analysis

**DOI:** 10.1186/s13023-020-1324-x

**Published:** 2020-02-04

**Authors:** Mohsen Jari, Reza Shiari, Omid Salehpour, Khosro Rahmani

**Affiliations:** 1 Department of Pediatric Rheumatology, Imam Hossein Children’s Hospital. Isfahan University of Medical Sciences, Isfahan, Iran; 2 Child Growth and Development Research Center, Research Institute for Primordial prevention of non-communicable Disease, Isfahan University of Medical Sciences, Isfahan, Iran; 3 Department of Pediatric Rheumatology, Shahid Beheshti University of Medical Sciences, Tehran, Iran; 4 Negah Specialty Ophthalmic Research Center, Shahid Beheshti University of Medical Sciences, Tehran, Iran

**Keywords:** Rheumatology, Pediatric, Uveitis, Treatment, Prevalence

## Abstract

**Background:**

Despite the low prevalence of uveitis in pediatric rheumatic diseases, potential problems as well as high disease burden can complicate its management. In this review, we systematically assessed the epidemiological, etiological, and managerial aspects of uveitis in pediatric rheumatic diseases.

**Methods:**

This current study was conducted in accordance with the established methods and the Preferred Reporting Items for Systematic Review and Meta-Analysis Protocols (PRISMA-P). We searched the manuscript databases, including Medline, Web of Knowledge, Google Scholar, Scopus, and Cochrane for all eligible studies in line with the considered keywords. We also conducted the statistical analysis using the Stata software.

**Results:**

Considering studies focusing on uveitis in Juvenile Idiopathic Arthritis (JIA) yielded a pooled prevalence of 11.8% (95%CI: 11.2 to 12.4%) for uveitis following JIA. In this regard, the prevalence rate of uveitis related to Behçet^,^s disease and Systemic Lupus Erythematosus (SLE( was estimated to be 15.0 and 0.8%, respectively. The pooled response rate to Adalimumab and Infliximab was estimated to be 68.0% (95%CI: 65.4 to 70.6%), 64.7% (95%CI: 59.8 to 69.3%), respectively. The documents for the systematical assessment of other biological medications (e.g. Tocilizumab, Daclizumab and Rituximab) were inadequate; however, the mean response rate for these drugs was 59, 75 and 80%, respectively. Our meta-analysis showed a pooled response rate of 40.0% (95%CI, 36.0% to 44.2) to Methotrexate. Significant heterogeneity and significant diffusion bias were demonstrated by reviewing studies.

**Conclusions:**

The pooled prevalence of uveitis in pediatric rheumatic diseases widely varied based on the underlying disease requiring more investigations in different subtypes of rheumatic diseases. The biologic medications, especially Adalimumab are the most effective treatments for uveitis in pediatric rheumatic diseases; however, a combination of the safe, available alternatives is preferred to achieve the most desirable treatment response.

## Background

Uveitis in pediatric rheumatic diseases is identified as an inflammatory event of the uvea of the iris, choroid, and retina. Although rheumatic diseases are partially common during childhood, the rheumatic disease-related uveitis is an uncommon finding in young people accounting for approximately5 to 10% of all individuals with uveitis [1, 2]. Despite its low prevalence, potential complications of uveitis as well as high disease burden present the disease management as a considerable challenge. Although uveitis, due to rheumatic diseases, may be easily diagnosed, the effective treatments of this event remain limited owing to serious systemic side effects [3]. More importantly, delay of diagnosis and treatment may lead to irreversible consequences like severe vision loss [4]. In this review, we systematically assessed the epidemiological, etiological and managerial aspects of uveitis in pediatric rheumatic diseases.

## Materials and methods

Search strategy: This study was conducted according to the previous established methods and in compliance with the Preferred Reporting Items for Systematic review and Meta-Analysis Protocols (PRISMA-P) [5]. The manuscript databases, including Medline, Web of Knowledge, Google Scholar, Scopus, and Cochrane were searched for any eligible studies in association with “Uveitis”, “Rheumatology”, and “pediatrics”. The studies were restricted to those written in English. The inclusion criteria were the epidemiology, etiologies, and the treatments of uveitis in pediatric rheumatic diseases. The exclusion criteria were introduced as follows: a lack of clear and reproducible results, non-English studies, lack of access to the full text manuscript, case reports, case series, and review papers.

Data abstraction and validity assessment: Data abstraction was independently performed by two un-blinded reviewers on the structure collection forms without divergences in data collection. The study quality was evaluated based on the following criteria: 1) the systematic review and meta-analysis based on the questions primarily described and formulated; 2) inclusion and exclusion criteria predefined in the studies as eligibility criteria; 3) searching the literature performed on a systematic and comprehensive approach; 4), the full texts of the article dually reviewed to minimize the bias 5) the quality of included studies independently rated by the reviewers for appraising internal validity 6) the comprehensive list of studies’ characteristics and findings7) the list of publication and risk of bias8) the assessment of heterogeneity [6]. The present study aimed to determine the global prevalence, causes and new therapies of rheumatoid arthritis in children by determining the prevalence as well as the odds ratio in the relationship between the major risk factors and disease risk. Furthermore, the year of publishing, number of included patients, and the method of design were pointed.

Statistical analysis: Dichotomous variables are reported as proportions and percentages, and continuous variables as mean values. Binary outcomes from individual studies were to be combined with both Mantel-Hansel fixed effect models. The odds ratio (OR) and 95% confidence interval (CI) were used as concise statistics to compare the dichotomous variables. Cochran’s Q test was used to determine the statistical heterogeneity of this study. This test was complemented with the I^2^ statistic quantifying the proportion of total variation across studies due to heterogeneity rather than chance. A value of I2 of 0–25% indicates insignificant heterogeneity, 26–50% low heterogeneity, 51–75% moderate heterogeneity, and 76–100% high heterogeneity. The publication bias was assessed by the rank correlation test and it was confirmed by the funnel plot analysis. The reported values were two-tailed, and hypothesis testing results were considered statistically significant at *p* = 0.05. Statistical analysis was conducted using the Stata software (version 13.1, Stata Corp, College Station, TX, USA).

## Results

### Prevalence and other epidemiological aspects of uveitis in pediatric rheumatic diseases

To assess different epidemiological aspects of uveitis in pediatric rheumatic diseases based on the applied keywords,19 out of 128 studies focused on the different epidemiological aspects of uveitis in pediatric rheumatic diseases and 4 were excluded due to lack of adequate information, and 2 were excluded due to lack of full text, or review article in nature. Finally, 13 studies published between 1997 and 2017 met the endpoints that were analyzed [7–19] (Table 1).

**Table 1:** The details of the studies on the prevalence and determinants of uveitis

Author, year	Number	M/F	Age at onset	Rheumatic dis.	Prevalence of Uveitis	Predictors of uveitis	Complications of uveitis
Nordal, 2017 [7]	435	149/286	5.5	JIA	89 (20.5)	age < 7 years at JIA onset AHA > 15 U/ml ANA	
Sardar, 2017 [8]	102	10/102	10.0	Behçet’s	15 (15.0)		synechiae, cataract, and macular edema
Kahwage, 2017 [9]	852	1/7	11.2	cSLE	7 (0.8)	Fever, lymphadenopathy	
Cecchin, 2017 [10]	274	50/224	11.5	JIA	57 (20.8)	Hypomobility	
Angeles, 2015 [11]	287	82/205	6.5	JIA	52 (18.0)	younger age oligoarticular subtype	Blindness
Angeles, 2013 [12]	4983		11.4	JIA	459 (11.6)	female sex early age of arthritis onset, oligoarticular subtype	
Clarke, 2013 [13]	79	42/37	9.0	JIA	18 (22.8)		
Shen, 2013 [14]	292	88/107	9.5	JIA	19 (6.7)		
Reininga, 2008 [15]	153			JIA	27 (17.6)		Visual loss, glaucoma, cataract, posterior synechiae, cystoid macular oedema and papillitis
Grassi, 2007 [16]	309	65/179	4.9	JIA	62 (20.1)	early age of arthritis onset ANA DRB1*11	
Heiligenhaus, 2005 [17]	3271			JIA	392 (12.0)	early age of arthritis onset female gender ANA	band keratopathy, posterior synechiae, cataract, glaucoma, and macula oedema
Chalom, 1997 [18]	760			JIA	74 (9.3)	early age of arthritis onset	synechiae, band keratopathy, cataract, or glaucoma
Akduman, 1997 [19]	78			JIA	7 (9.0)		Visual loss band keratopathy and cataract

**Fig. 1:**
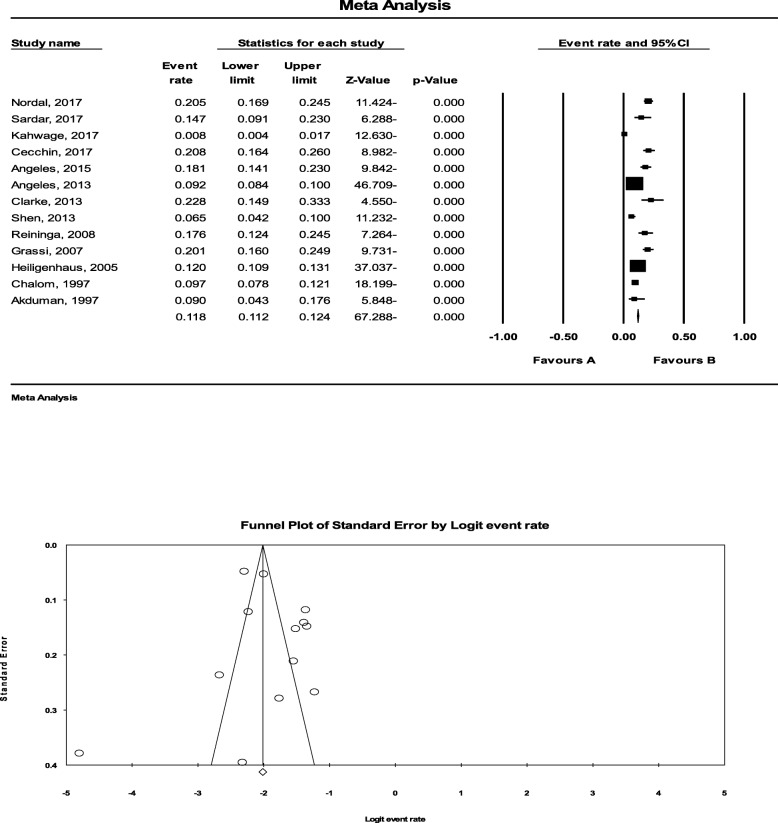
Prevalence of JIA-related uveitis. Our meta-analyses yielded a pooled prevalence of11.8% (95%CI: 11.2 to 12.4%) for uveitis following JIA. To determine overall prevalence of JIA-related uveitis, the statistical heterogeneity was significant with an I^2^ of 93.771% (*P* < 0.001)

In total, 11,875 patients were assessed indicating a higher rate of uveitis in female than in male children. The average age of the patients at the beginning of uveitis was 8.8 years. Of 13 patients focused uveitis in pediatric rheumatic diseases, most of them (11 out of 13 manuscripts) included children suffering Juvenile Idiopathic Arthritis (JIA), while Behçet^,^s disease-related uveitis was assessed in one study and Systemic Lupus Erythematosus (SLE-related) uveitis in another. Initially considering studies focused on JIA-related uveitis yielded a pooled prevalence of11.8% (95%CI: 11.2 to 12.4%) for uveitis following JIA (Fig. 1). In this regard, the prevalence rate of uveitis related to Behçet^,^s disease and SLE was estimated to be 15.0 and 0.8%, respectively. The main predictors of uveitis in children suffering from rheumatic diseases were female gender, age < 7 years at the onset of JIA (particularly in girls), oligoarticular subtype of disease and positive antinuclear antibody (ANA) > 15 U/ml. ANA is positive in 70 to 90% those with uveitis. In this regard, poly-articular RF-positive subtype of JIA was revealed to be protective of uveitis. Reviewing the literature showed a strong racial tendency to uveitis in pediatric rheumatic diseases, so that the possibility of JIA-related uveitis in caucasian white children was nearly twice as much as African-American children. Regarding uveitis-related complications, the common complications encompassed band keratopathy (15.7 to 29%), synechiae (27 to 33%), cataract (8 to 31%), macular edema (6 to 25%), ocular hypertension/glaucoma (8 to 19%), and macular fibrosis (4%). Overall, complications of uveitis developed in 35.5 to 67% of children that one-third of them were present at diagnosis. Final visual acuity less than 20/50 was found in 11 to 31% and less than 20/200 in 12% of eyes, but blindness widely occurred from 0 to 17.5% in the affected children that was more common in African American children than in Caucasian children. The risk to vision is higher if JIA begins in the preschool years. To determine the overall prevalence of JIA-related uveitis, the statistical heterogeneity was significant with an I^2^ of 93.771% (*P* < 0.001) (Fig. 1). There was a significant publication bias as evidenced by either funnel plot asymmetry or the Egger test (*P* = 0.026).

### Pathophysiology of uveitis in pediatric rheumatic diseases

At the beginning of the last century, the eye manifestations of rheumatic diseases, especially in children with JIA had been well described. Since then, numerous cases of JIA-related uveitis have been reported. Formerly, many cases might have been missed in diagnosis, particularly asymptomatic patterns leading to high rates of visual loss; however, by advancing diagnostic approaches, this event is now rarely reported. The nature of rheumatic disease-related uveitis is mostly insidious at the onset and in some cases may be followed by a remitting course. Both eyes are mostly affected. In many cases, uveitis is non-granulomatous with a faint flare. In mild cases, Keratic precipitates can be seen in the inferior half of the corneal endothelium progressed to the anterior vitreous in severe cases with spreading inflammation to the posterior parts of the eye. Most changes have been identified to be linked to Human Leukocyte Antigen B27 (HLA B27) as one of the main markers for arthritis.

Naturally, uveitis is more commonly discovered in children with oligoarthritis and more rarely in systemic-onset arthritis. JIA-related uveitis more commonly occurs earlier in females than in males, a difference, which has not been exclusively explained. Although 90% of uveitis cases are revealed within the first 4 years of arthritis, it may sometimes occur in the first 7 years after onset of arthritis.

The pathogenesis of JIA and its associated uveitis is unknown. It is presumed to be autoimmune in nature. Genetically, histocompatibility allele profiles have been widely assessed, and higher expression of some HLA alleles such as DRB1*1104 andDRB1*01 was found in those with uveitis emphasizing the autoimmune nature of disease. Furthermore, the positivity of ANA in most cases also emphasizes this fact. Additionally, immune complex deposition has a potential place in the pathogenesis of uveitis, but the details of autoimmunity and specific autoantibodies in pathogenesis of uveitis are under investigation.

### Recent advances in the treatment of uveitis in pediatric rheumatic diseases

Table 2 summarizes different medical therapeutic approaches to uveitis in pediatric rheumatic diseases. Formerly, two groups of drugs, including glucocorticoids and nonbiologic Disease-Modifying Anti Rheumatic Drugs (DMARDS), were widely used to improve uveitis; however, by developing biological agents, these medications have been considered particularly. To evaluate the efficacy, response rates and complications of any old and novel drugs based on the applied keywords, of total 157 studies initially conducted, 42 studies published between 1998 and 2017 focused on different medications against uveitis (Table 2. At the end of document text file).

**Table 2:** The details of the studies on the response to different therapeutic regimens

Author, year	Type of study	Disease	Number	Medication	Response rate
Correll, 2017 [20]	Review chart	JIA	60	Adalimumab	80.0%
Horneff, 2016 [21]	Review chart	JIA	236	Adalimumab	61.0%
Castiblanco, 2016 [22]	Review chart	JIA	14	Adalimumab	77.0%
Henderson, 2016 [23]	cohort study	JIA	92	Adalimumab	68.0%
Klotsche, 2016 [24]	cohort study	JIA	320	Adalimumab	77.0%
Schmeling, 2014 [25]	Review chart	JIA	289	Adalimumab	63.4%
García, 2013 [26]	Clinical trial	JIA	39	Adalimumab	60.0%
Lerman, 2013 [27]	Review chart	JIA	56	Adalimumab	75.0%
Simonini, 2013 [28]	Clinical trial	JIA, Behçet’s	14	Adalimumab	57.4%
Zannin, 2013 [29]	Cohort Study	JIA	108	Adalimumab	67.4%
Trachana, 2011 [30]	Cohort Study	JIA	9	Adalimumab	65.4%
Tynjälä, 2008 [31]	Review chart	JIA	20	Adalimumab	65.0%
Gallagher, 2007 [32]	case series	JIA	23	Adalimumab	77.0%
Vazquez, 2006 [33]	Clinical trial	JIA	9	Adalimumab	80.8%
Sardar, 2017 [8]	Review chart	JIA	56	Infliximab	80.0%
Aeschlimann, 2017 [34]	Review chart	JIA	52	Infliximab	60.0%
Aeschlimann, 2014 [35]	Review chart	JIA	82	Infliximab	57.0%
Tambralli, 2013 [36]	Review chart	JIA	95	Infliximab	74.1%
Zannin, 2013 [29]	Review chart	JIA	48	Infliximab	42.8%
Tugal, 2008 [37]	Review chart	JIA	20	Infliximab	80.0%
Ardoin, 2007 [38]	Case series	JIA	16	Infliximab	79.0%
de Oliveira, 2007 [39]	Review chart	JIA	30	Infliximab	70.0%
Tynjälä, 2007 [40]	Review chart	JIA	21	Infliximab	31.0%
Rajaraman, 2006 [41]	Review chart	JIA	6	Infliximab	100%
Saeed, 2014 [42]	Review chart	JIA	9	Etanercept	66.7%
Foeldvari, 2007 [43]	Review chart	JIA	34	Etanercept	70.0%
Tynjälä, 2007 [40]	Review chart	JIA	45	Etanercept	31.0%
de Oliveira, 2007 [39]	Review chart	JIA	9	Etanercept	70.0%
Horneff, 2016 [21]	Review chart	JIA	94	Etanercept	68.0%
Quesada, 2017 [44]	Review chart	JIA	89	Tocilizumab	46.0%
Horneff, 2016 [21]	Review chart	JIA	74	Tocilizumab	61.0%
Tappeiner, 2016 [45]	Review chart	JIA	17	Tocilizumab	58.8%
Miserocchi, 2016 [46]	Review chart	JIA	8	Rituximab	75.0%
Gallagher, 2007 [32]	Review chart	JIA	23	Daclizumab	80.0%
Henderson, 2016 [23]	cohort study	JIA	92	Methotrexate	76.0%
Saeed, 2014 [42]	Review chart	JIA	147	Methotrexate	34.0%
Marvillet, 2009 [47]	Review chart	JIA	75	Methotrexate	17.4%
Papadopoulou, 2013 [48]	Review chart	JIA	254	Methotrexate	33.9%
Kalinina, 2011 [49]	Review chart	JIA	22	Methotrexate	82.0%
Heiligenhaus, 2007 [50]	Review chart	JIA	31	Methotrexate	41.9%
Shetty, 1999 [51]	Review chart	JIA	4	Methotrexate	50.0%
Weiss, 1998 [52]	Review chart	JIA	7	Methotrexate	85.7%

**Fig. 2:**
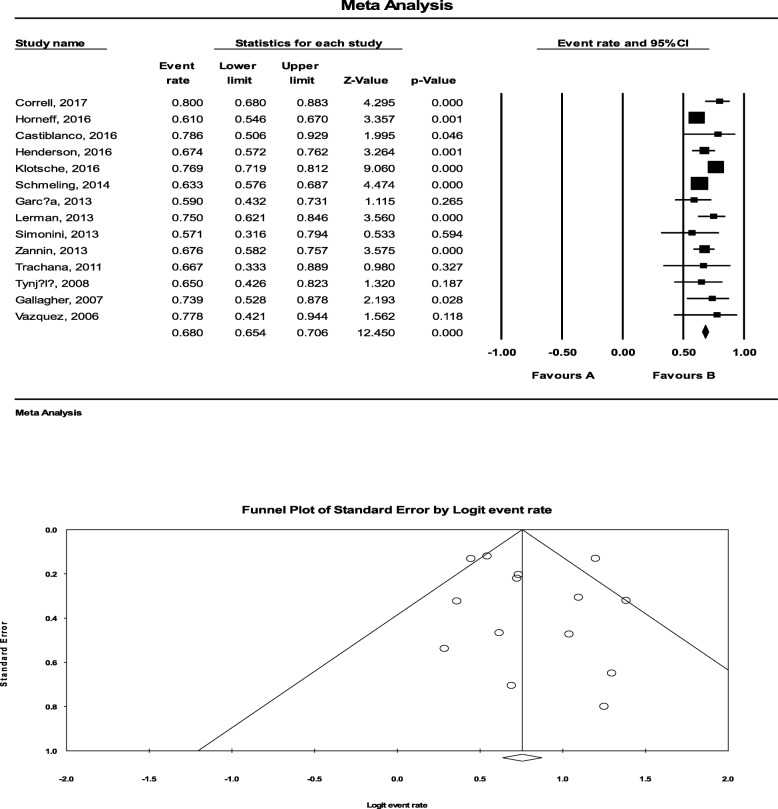
The efficacy of Adalimumab to treat JIA-related uveitis. The efficacy of Adalimumab was assessed in 1289 patients. The pooled response rate to Adalimumab was estimated to be 68.0% (95%CI: 65.4 to 70.6%). Assessment of the studies on the efficacy of Adalimumab indicated that the statistical heterogeneity was significant with an I^2^ of 57.164%

Of 42 studies, 34 focused on biological agents (Adalimumab in 14 studies, Infliximab in 10 studies, Etanercept in 5 studies, Tocilizumab in 3 studies, Rituximab in 1 study and Daclizumab in 1 study). In addition, Methotrexate as a common used DMARD for uveitis was assessed in 8 studies. Totally, the efficacy of Adalimumab was assessed in 1289 patients. The pooled response rate to Adalimumab was estimated to be 68.0% (95%CI: 65.4 to 70.6%). The drug-related side effects were recorded in the wide range of 3 to 52%, including non-ocular complications (e.g. autoimmune diseases, local pain, anemia, depression, abscess and even sepsis) and ocular complications (e.g. cataracts, and uveitis flares). However, they were mostly tolerable with the patients. In assessing the studies on the efficacy of Adalimumab, the statistical heterogeneity was significant with an I^2^ of 57.164% (*P* = 008) (Fig. 2). There was no significant publication bias as evidenced by either funnel plot asymmetry or the Egger test (*P* = 0.576).

**Fig. 3:**
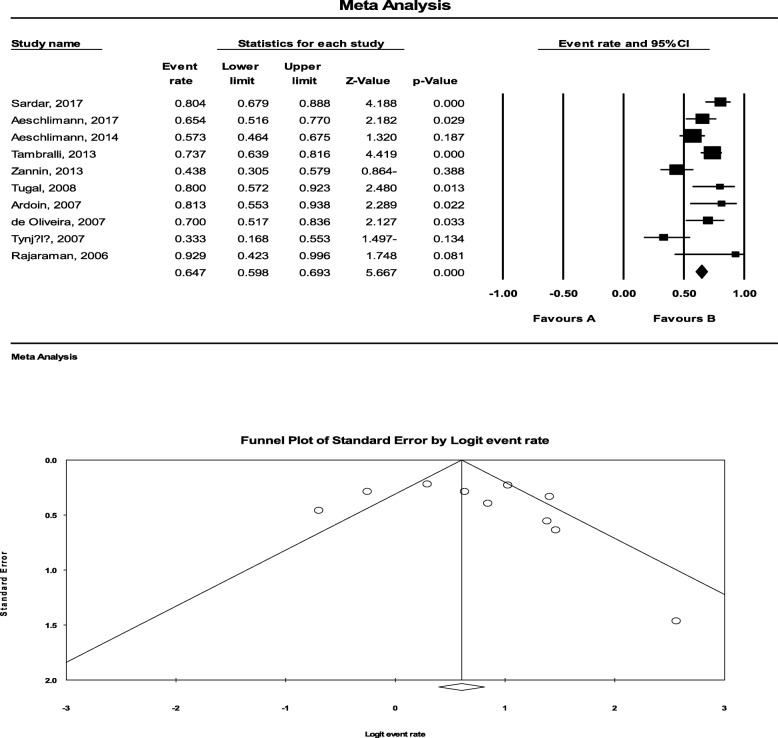
The efficacy of Infliximab to treat JIA-related uveitis. To determine the efficacy of Infliximab, 476 patients were tested for the medications leading to a pooled response rate of 64.7% (95%CI: 59.8 to 69.3%). In this regard, the statistical heterogeneity was also significant with an I^2^of 73.066% (*P* < 0.001). There was no significant publication bias (*P* = 234)

To determine the efficacy of Infliximab, 476 patients were examined for the medications leading to a pooled response rate of64.7% (95%CI: 59.8 to 69.3%). The most common side effects of the medications were the reactivation of uveitis and infusion reaction in approximately two-third of the patients, infectious events, vitreous hemorrhage, and systemic infections. In this regard, the statistical heterogeneity was also significant with an I^2^ of 73.066% (*P* < 0.001) (Fig. 3). There was no significant publication bias (*P* = 234).

**Fig. 4:**
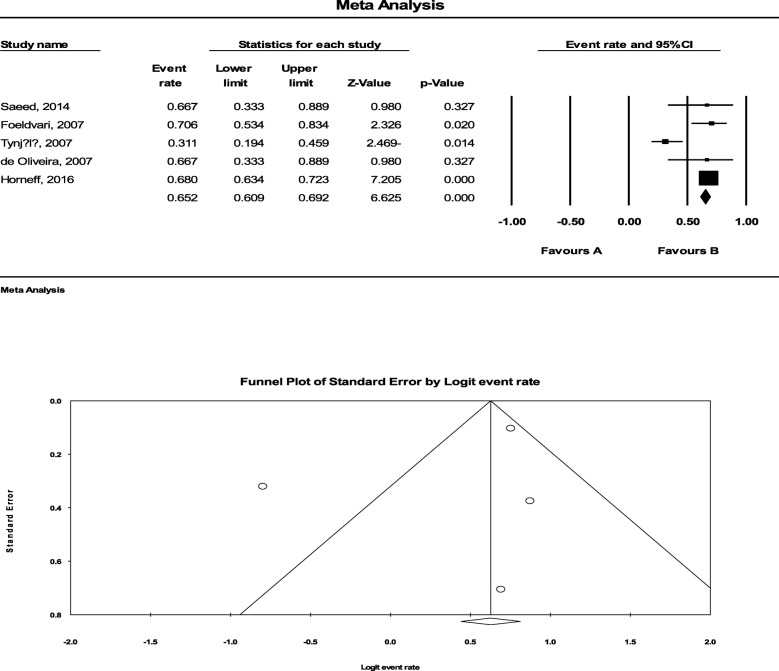
The efficacy of Etanercept to treat JIA-related uveitis. Respecting the efficacy of Etanercept, 516 patients were examined for this drug leading to pooled drug efficacy in 65.2% (95%CI: 60.9 to 69.2%). The statistical heterogeneity was significant with an I^2^ of 81.342% (*P* < 0.001). There was also no significant publication bias (*P* = 0.234)

Given the efficacy of Etanercept, 516 patients were examined for this drug leading to pooled drug efficacy in 65.2% (95%CI: 60.9 to 69.2%). The most common side effects of this drug were infections and in some cases drugs intolerability. The documents to systematically assess other biological medications such as Tocilizumab and Rituximab were inadequate, but the mean response rates for these drugs were 59 and 75%, which required more investigation. The statistical heterogeneity was significant with an I^2^ of 81.342% (*P* < 0.001) (Fig. 4). There was also no significant publication bias (*P* = 0.234).

**Fig. 5:**
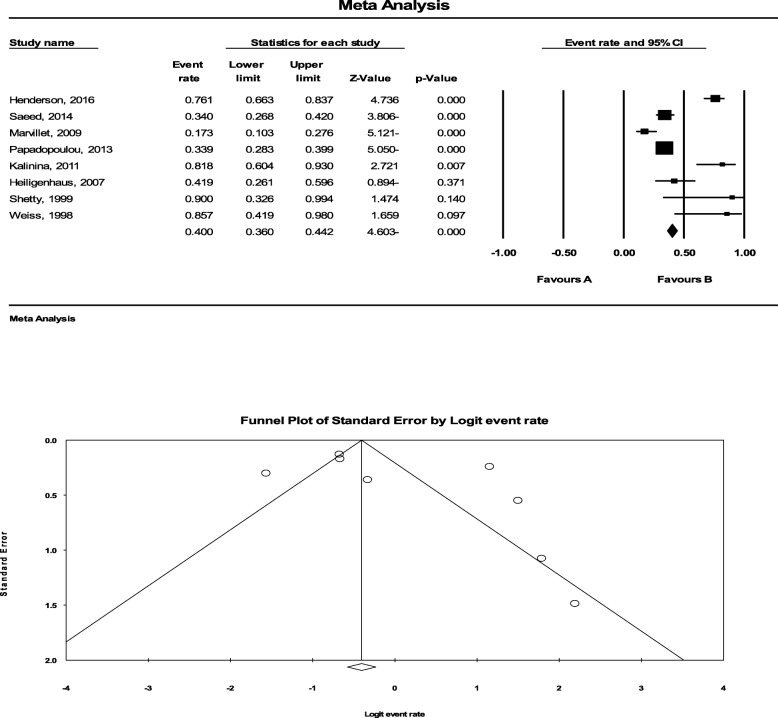
The efficacy of Methotrexate to treat JIA-related uveitis. To determine the efficacy of Methotrexate, we systematically reviewed 8 studies consisted of 632 patients and could show a pooled response rate of40.0% (95%CI: 36.0% to 44.2) to Methotrexate. The statistical heterogeneity was also significant with an I^2^ of 91.314% (*P* < 0.001). There was also a significant publication bias (*P* = 0.016)

Of DMARDs, only Methotrexate was exclusively evaluated. In this regard, we systematically reviewed 8 studies consisted of 632 patients and could show a pooled response rate of40.0% (95%CI: 36.0% to 44.2) to Methotrexate. The statistical heterogeneity was also significant with an I^2^ of 91.314% (*P* < 0.001) (Fig. 5). There was also a significant publication bias (*P* = 0.016).

## Discussion

In our systematic review, we attempted to consider uveitis sourced from all types of pediatric rheumatic diseases. Nevertheless, previous studies mostly focused on children with JIA, which is the most frequent cause for uveitis and the most important rheumatic disease with extra-articular signs. Epidemiologically, owing to differences in the type of studies, the geographical area as well as the different criteria for determining uveitis between 11.6 and 30.0% [53, 54], the prevalence of uveitis associated with JIA was varied. However, our meta-analyses yielded a pooled prevalence of 11.8% (95%CI: 11.2 to 12.4%) for uveitis following JIA, which is close to the lower limit of the prevalence published in the literature. There seems to be a decreasing incidence of uveitis due to early detection and selection of appropriate, preventive, and therapeutic regimens. In the current systematic review, we also examined potential risk factors for uveitis in pediatric rheumatic disease: female gender, age < 7 years at the onset of JIA (particularly in girls), oligoarticular subtype of JIA and positive ANA. To put it differently, a combination of both clinical and laboratory markers can be employed to predict the possibility of uveitis in pediatric rheumatic diseases. In the literature, the main indicators for uveitis include female gender, category of JIA, particularly oligoarticular disease, younger age of onset, positive ANA, and HLA-B27 [55, 56]. It should be noted that the role of ethnicity in predicting uveitis remained uncertain. Given the clinical features and complications of childhood rheumatoid uveitis, keratopathy, synechiae, cataract, macular edema, ocular hypertension/glaucoma, and macular fibrosis were the main clinical features of this disease. In general, between one-third and two-third of patients suffer from these manifestations, albeit in cases with delayed diagnosis, severe visual loss and even blindness are expected.

The development of new biological drugs could make JIA-associated uveitis a controllable and early-diagnosed disease over the last decade. Particularly, introduction of TNF-inhibitors could successfully control disease poor prognosis. Our review had the highest concentrations of Adalimumab and Infliximab in the management of uveitis in these patients with a response rate of 68.0 and 64.7%, which appeared to be somewhat acceptable. In this regard, it seems that a combination of biological drugs with other subgroups of drugs such as DMARDs and even glucocorticoids may have been beneficial to achieve a complete recovery under these drugs. The potential side effects and clinical limitations of such drugs should also be considered. Although this review could show higher response rates to some other biological-based drugs such as Tocilizumab and Rituximab as 59 and 75%, respectively, a few studies focused on the effectiveness of these drugs; therefore, the clinical efficacy and their potential side effects were unclear. In general, the results demonstrated the high efficacy and safety of biological agents, especially Adalimumab.

As an important finding, reviewing the studies assessed the efficacy of biological agents reaching the overall responses with a low standard deviation and indicating the correctness of the study design, drug dosages used, and homogeneity of sampling selected for the studies. However, in evaluated studies of DMARDs, especially methotrexate, various responses to medications (33.9 to 85.7%) have been reported. In fact, this variety of response rate might be a reason for the low efficacy of these types of drugs for pediatrics. In addition, the necessity for discontinuing drugs and changing them to other drug families may be considered. We could show significant publication bias in those studies focused on DMARDs. The publication bias in medical journals refers to the publication of more articles containing positive conclusions or significant statistical results. This bias suggests that articles containing negative or non-significant statistical results are less likely to be published. The first cause of this bias is that the researchers themselves do not intend to report their negative or non-significant statistical results. Moreover, some organizations that provide funding for medical research may refuse to publish such findings or, at least, delay publication. Thus, the results published on the efficacy of some drugs such as DMARDs on pediatric uveitis might be unreliable, as they require predesign and pre-implementation.

## Conclusion

In this systematic review, we attempted to consider uveitis originated from all types of pediatric rheumatic diseases. However, JIA, especially oligoarticular subtype of disease (positive ANA). is the most frequent cause of uveitis. This study showed the highest efficacy of Adalimumab and Infliximab in the management of uveitis. The efficacy of some drugs such as Tocilizumab and Rituximab showed moderate to high responses, respectively, and few studies focused on the effectiveness of these drugs; therefore, the clinical efficacy and their potential side effects were unclear. In general, the results summarize the efficacy and safety of biological agents, particularly Adalimumab. In the evaluated studies of DMARDs, especially methotrexate, mild to moderate responses have been reported.

## Data Availability

The data are available on request to the corresponding author.

## Abbreviations

ANA: Antinuclear antibody
CI: Confidence Interval
DMARDS: Disease Modifying Anti-Rheumatic Drugs
HLA: Human Leukocyte Antigen
JIA: Juvenile Idiopathic Arthritis
OR: Odds Ratio
PRISMA-P: Preferred Reporting Items for Systematic review and Meta-Analysis Protocols
SLE: Systemic Lupus Erythematosus
